# Effect of an Automated Patient Dashboard Using Active Choice and Peer Comparison Performance Feedback to Physicians on Statin Prescribing: The PRESCRIBE Cluster Randomized Clinical Trial

**DOI:** 10.1001/jamanetworkopen.2018.0818

**Published:** 2018-07-27

**Authors:** Mitesh S. Patel, Gregory W. Kurtzman, Sneha Kannan, Dylan S. Small, Alexander Morris, Steve Honeywell, Damien Leri, Charles A. L. Rareshide, Susan C. Day, Kevin B. Mahoney, Kevin G. Volpp, David A. Asch

**Affiliations:** 1 Penn Medicine Nudge Unit, University of Pennsylvania, Philadelphia; 2 Perelman School of Medicine, University of Pennsylvania, Philadelphia; 3 The Wharton School, University of Pennsylvania, Philadelphia; 4 Center for Health Incentives and Behavioral Economics, University of Pennsylvania, Philadelphia; 5 Penn Medicine Center for Health Care Innovation, University of Pennsylvania, Philadelphia; 6 Corporal Michael J. Crescenz Veterans Affairs Medical Center, Philadelphia, Pennsylvania; 7 Department of Medicine, Massachusetts General Hospital, Boston

## Abstract

**Importance:**

Statins are not prescribed to approximately 50% of patients who could benefit from them.

**Objective:**

To evaluate the effectiveness of an automated patient dashboard using active choice framing with and without peer comparison feedback on performance to nudge primary care physicians (PCPs) to increase guideline-concordant statin prescribing.

**Design, Setting, and Participants:**

This 3-arm cluster randomized clinical trial was conducted from February 21, 2017, to April 21, 2017, at 32 practice sites in Pennsylvania and New Jersey. Participants included 96 PCPs and 4774 patients not previously receiving statin therapy. Data were analyzed from April 25, 2017, to June 16, 2017.

**Interventions:**

Primary care physicians in the 2 intervention arms were emailed a link to an automated online dashboard listing their patients who met national guidelines for statin therapy but had not been prescribed this medication. The dashboard included relevant patient information, and for each patient, PCPs were asked to make an active choice to prescribe atorvastatin, 20 mg, once daily, atorvastatin at another dose, or another statin or not prescribe a statin and select a reason. The dashboard was available for 2 months. In 1 intervention arm, the email to PCPs also included feedback on their statin prescribing rate compared with their peers. Primary care physicians in the usual care group received no interventions.

**Main Outcomes and Measures:**

Statin prescription rates.

**Results:**

Patients had a mean (SD) age of 62.4 (8.3) years and a mean (SD) 10-year atherosclerotic cardiovascular disease risk score of 13.6 (8.2); 2625 (55.0%) were male, 3040 (63.7%) were white, and 1318 (27.6%) were black. In the active choice arm, 16 of 32 PCPs (50.0%) accessed the patient dashboard, but only 2 of 32 (6.3%) signed statin prescription orders. In the active choice with peer comparison arm, 12 of 32 PCPs (37.5%) accessed the patient dashboard and 8 of 32 (25.0%) signed statin prescription orders. Statins were prescribed in 40 of 1566 patients (2.6%) in the usual care arm, 116 of 1743 (6.7%) in the active choice arm, and 117 of 1465 (8.0%) in the active choice with peer comparison arm. In the main adjusted model, compared with usual care, there was a significant increase in statin prescribing in the active choice with peer comparison arm (adjusted difference in percentage points, 5.8; 95% CI, 0.9-13.5; *P* = .008), but not in the active choice arm (adjusted difference in percentage points, 4.1; 95% CI, −0.8 to 13.1; *P* = .11).

**Conclusions and Relevance:**

An automated patient dashboard using both active choice framing and peer comparison feedback led to a modest but significant increase in guideline-concordant statin prescribing rates.

**Trial Registration:**

ClinicalTrials.gov Identifier: NCT03021759

## Key Points

**Question:**

Can an automated patient dashboard and nudges to physicians increase guideline-appropriate statin prescription rates among patients who were not previously receiving statin therapy?

**Findings:**

In this cluster randomized clinical trial of 96 primary care physicians from 32 practice sites including 4774 patients, an automated patient dashboard using active choice framing and peer comparison feedback led to a modest but significant increase in guideline-appropriate statin prescribing rates compared with usual care.

**Meaning:**

Nudges to physicians offer an effective, low-cost, and scalable approach to increase use of automated patient dashboards to improve guideline-concordant prescribing behaviors, but these approaches may need to be designed to better fit within clinician workflow or be combined with other approaches to further increase their impact.

## Teaser

This 3-arm cluster randomized clinical trial evaluates the effectiveness of an automated patient dashboard using active choice framing with and without peer comparison feedback on performance to nudge primary care physicians to increase guideline-concordant statin prescribing.

## Introduction

Atherosclerotic cardiovascular disease (ASCVD) is the leading cause of morbidity and mortality in the United States.^1,2^ Statins, or 3-hydroxy-3-methylglutaryl coenzyme A reductase inhibitors, are generally well tolerated and have been demonstrated to lower the risk of cardiovascular events and mortality by approximately 30%.^3,4,5^ However, despite their clinical utility and established national guidelines,^3,6^ clinicians fail to prescribe them to approximately 50% of patients who could benefit from them.^7,8,9,10,11^

The traditional approach for clinicians to identify patients eligible for statins is by individually recognizing that a patient meets clinical criteria. Even when systems are automated, reminders are typically provided at the time of a visit. This approach often leads to delays or failures in care because some patients visit their physician infrequently and because preventive services may not be discussed if there are acute issues or limited time.

An automated dashboard of patients eligible for statin therapy could address many of these challenges. Dashboards could help clinicians identify gaps in care for an entire panel of patients, untethered from the timing of individual visits. Many health systems are already using such dashboards to provide feedback to clinicians on variations in care. However, the association of these dashboards with patient outcomes has not been well examined. A key challenge in this approach is that clinicians must both use the dashboard and act on it for it to lead to improvements in health.

Nudges could be targeted to clinicians to engage them in using these dashboards to improve the delivery of care.^12,13^ For example, clinicians could be prompted to review a list of eligible patients and make an active choice whether or not to prescribe statins. Active choice is a method used to address delays in decision making by prompting an immediate decision between alternative choices.^13,14,15^ In previous observational studies,^16,17^ we demonstrated how active choice delivered to physicians through the electronic health record (EHR) during patient interactions significantly increased ordering of influenza vaccination, colonoscopy, and mammography. In addition, health systems could deliver feedback to clinicians informing them how their performance compares with other peers in their network,^18^ which has been used in a variety of contexts to motivate better performance, including reducing inappropriate antibiotic prescribing.^19,20,21^

In this cluster randomized clinical trial, our objective was to evaluate the effectiveness of an automated patient dashboard using active choice framing with and without peer comparison feedback on performance to nudge primary care physicians (PCPs) to prescribe guideline-appropriate statins for patients who were not previously receiving statin therapy. The trial was implemented in a pragmatic fashion to inform health systems’ efforts to redesign approaches to better manage populations of patients.

## Methods

### Study Design

The Pragmatic Randomized Evaluation of Statin Active Choice to Reach Improved Outcomes Based on Evidence (PRESCRIBE) was a 3-arm cluster randomized clinical trial conducted among PCPs from 32 clinics at the University of Pennsylvania Health System (UPHS). The trial was conducted during a 2-month period between February 21, 2017, and April 21, 2017, and compared new statin prescription rates for usual care with PCPs receiving an active choice intervention with and without peer comparison performance feedback. The study protocol was approved by the University of Pennsylvania institutional review board. The trial protocol can be found in Supplement 1. Informed consent by physicians and patients was waived because this was a pragmatic evaluation of a health system initiative that posed minimal risk. Neither physicians nor patients were compensated for participation. This study followed the Consolidated Standards of Reporting Trials (CONSORT) reporting guideline.

### Study Sample

Data on PCPs from the UPHS and their patients were obtained from the EHR using the reporting database Clarity (Epic). The PCPs at the UPHS were eligible if they had at least 10 patients who met the 2013 American College of Cardiology/American Heart Association guidelines for statin therapy,^3^ including any form of clinical ASCVD, most recent low-density lipoprotein cholesterol (LDL-C) level of 190 mg/dL (to convert to millimoles per liter, multiply by 0.0259) or greater, age 40 to 75 years with diabetes and LDL-C level of 70 to 189 mg/dL, and age 40 to 75 years with an estimated 10-year ASCVD risk score^22^ of 7.5% or greater. Nurse practitioners and resident physicians were excluded.

Patients were eligible if they had a PCP at the UPHS, met the 2013 American College of Cardiology/American Heart Association guidelines for statin therapy,^3^ and had no documentation of a previous statin prescription in our EHR. Patients were excluded if they had stage 4 or 5 chronic kidney disease with a glomerular filtration rate (GFR) of 30 mL/min or less, a documented allergy to statins, or previous adverse reaction to statins, including myopathy, rhabdomyolysis, or hepatitis.

### Randomization

The PCPs were electronically randomized to a study arm using block sizes of 3. Randomization was stratified on quartile of PCP baseline statin prescribing rates before entry into the trial to ensure both a range of baseline PCP prescribing rates within each arm and a balance across study arms. All investigators, statisticians, and data analysts were blinded to arm assignments until the study and analysis were completed.

### Interventions

The PCPs and patients in usual care received no communications or interventions from the trial. In both active choice intervention arms, each PCP received an email from the study team that indicated the number of his or her patients who met guidelines for statin therapy but had not been prescribed a statin. The email provided the PCP with a link to a dashboard on a secure website that listed each of these patients along with their age, sex, and the following data as available from the EHR: 10-year ASCVD risk score, most recent LDL-C level, other lipid levels (ie, total cholesterol, high-density lipoprotein, and triglycerides), body mass index, history of smoking and any form of clinical ASCVD (eg, myocardial infarction or stroke), liver function tests (normal or slightly elevated), and medical record number (for EHR lookup if warranted). The PCPs were asked to review the list of patients within 1 week and use the dashboard to select whether or not to prescribe each patient a statin. The dashboard provided an overview of the study, a link to the American College of Cardiology/American Heart Association guidelines, and options for selecting statin dosage (eFigure 1 and eFigure 2 in Supplement 2). The PCPs could either click a button at the top of the dashboard that would select atorvastatin, 20 mg, once daily for all patients or individually review and select choices for each patient from among the following 4 options: (1) atorvastatin statin, 20 mg, once daily; (2) atorvastatin at another dose; (3) another statin (choice of simvastatin, pravastatin, or rosuvastatin doses); or (4) don’t prescribe, in which case the PCP had to select 1 of the following reasons: patient not eligible, patient declined, adverse effect or allergy not listed in Epic, drug interaction, risks outweigh benefits, or other.

In the peer comparison arm, the email to PCPs also included performance feedback on their baseline statin prescribing rates prior to entry in the trial compared with their peers (eFigure 3 and eFigure 4 in Supplement 2). The PCPs below the median were informed of how they compared with the median (eg, your statin prescribing rate: 50%; average of your peers at Penn: 64%). The PCPs above the median but below the 90th percentile were informed how they compared with the 90th percentile or top performers (eg, your statin prescribing rate: 70%; your top-performing peers at Penn: 85%). The PCPs at or above the 90th percentile were not given comparison data and instead informed of their high performance (among your peer physicians at Penn, you are a top performer. Great job!). This design was based on evidence that social norming interventions are more effective when combined with an injunctive norm that infers a social approval or disapproval.^23^ The tiered approach provides 90% of PCPs with comparisons with others with better performance (rather than sending everyone a comparison with the median), which may encourage behavior toward improvement and prevent regression to the mean or lowest performer.^24^

The PCPs in the intervention arms received up to 2 email reminders over the next 2 weeks, and the dashboard remained available for 2 months. The PCPs could save their work and return later or finalize and submit selections. Once submitted, a study coordinator templated an order for the medication in the EHR and sent the PCP an email when the prescription was ready to be electronically signed. Once the prescription was signed, the study coordinator mailed the patient a letter informing him or her of the PCP’s decision to prescribe a statin, the location of the pharmacy to which the prescription had been sent, the risks and benefits of statins, and a recommendation for a follow-up visit with the PCP within 6 months (eFigure 5 in Supplement 2).

### Outcome Measures

The primary outcome was the change in the percentage of eligible patients prescribed a statin. This measure was evaluated during the 2-month intervention period.

### Statistical Analysis

A priori power calculations were based on an initial review of existing EHR data that revealed a 65% baseline statin prescribing rate, a median of 38 eligible patients per PCP but not yet prescribed a statin, and an intracluster correlation for patients prescribed a statin within a PCP panel of 0.026. Based on these assumptions, we estimated that a sample of 84 PCPs (28 per arm) would provide at least 90% power to detect a 10% difference between each of the arms using a conservative Bonferroni adjustment of the type I error rate with a 2-sided α of .017. We increased the PCP enrollment target to 96 to account for a potential 15% dropout rate.

All randomly assigned PCPs and their patients were included in the intention-to-treat analysis. For each patient, we used the EHR to identify whether a statin had been prescribed by the end of the 2-month study period. In the main adjusted analysis, we used the PROC GENMOD feature in SAS (SAS Institute Inc) to fit models based on generalized estimating equations with a logit link and an independence correlation structure using PCP as the clustering unit.^25^ To test the robustness of our findings, we conducted a sensitivity analysis in which the main model was estimated by adjusting for patient characteristics, including age, sex, race/ethnicity, insurance type, median annual household income by zip code, Charlson comorbidity index score,^26^ body mass index, most recent LDL-C level measurement, 10-year ASCVD risk score,^22^ clinical ASCVD, congestive heart failure, diabetes, hypertension, and smoking history, as well as PCP panel size. In post hoc exploratory analyses, the sensitivity model was further adjusted for PCP degree and using interaction terms of the study arm variables with race/ethnicity and separately with insurance type.

To obtain the adjusted difference and 95% confidence intervals in the percentage of patients prescribed a statin between arms, we used the bootstrap method,^27,28^ resampling patients 1000 times. Resampling of patients was conducted by a PCP panel to maintain clustering at the PCP level. All analyses were conducted using SAS, version 9.4 (SAS Institute Inc).

## Results

Ninety-six PCPs from 32 primary care clinics and 4774 patients were randomized (Figure). The PCPs differed slightly across arms for type of medical degree, but all other characteristics were well balanced (Table 1). The mean (SD) baseline statin prescribing percentage before the trial was 63.5% (10.3%), and a mean (SD) of 49.7 (41.0) patients were enrolled per PCP. The patient sample had a mean (SD) age of 62.4 (8.3) years, 2625 (55.0%) were male, 3040 (63.7%) were white, and 1318 (27.6%) were black. The mean (SD) 10-year ASCVD risk score was 13.6 (8.2); 2769 (58.0%) had hypertension, 2049 (42.9%) had a history of smoking, and 1201 (25.2%) had diabetes. Patients differed slightly across arms in a few sociodemographic and baseline characteristics but were mostly well balanced (Table 2).

**Figure.**
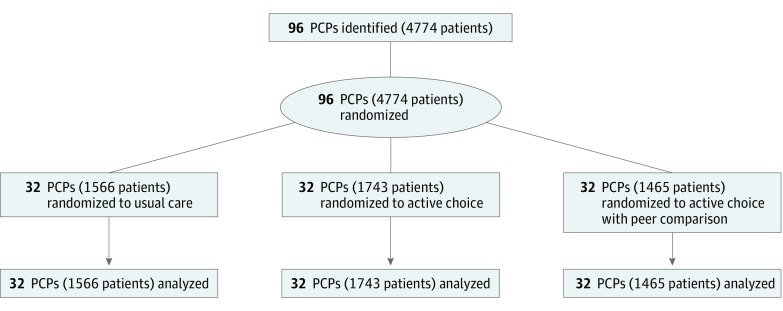
CONSORT Diagram Primary care physicians (PCPs) were randomly assigned with their patients to an arm for a 2-month study period. No patients were lost to follow-up.

**Table 1.** Characteristics of the Physician Sample

Characteristic	Usual Care (n = 32)	Active Choice (n = 32)	Active Choice With Peer Comparison (n = 32)
Male, No. (%)	13 (40.6)	19 (59.4)	16 (50.0)
Years in practice, mean (SD)	22.7 (11.9)	20.9 (12.4)	20.4 (11.5)
Medical degree, No. (%)			
Doctor of medicine	26 (81.3)	29 (90.6)	32 (100.0)
Doctor of osteopathic medicine	6 (18.8)	3 (9.4)	0 (0.0)
Medical specialty, No. (%)			
Family medicine	15 (46.9)	15 (46.9)	11 (34.4)
Internal medicine	17 (52.1)	17 (52.1)	21 (65.6)
Baseline measures, mean (SD)			
Patients enrolled in trial, No.	48.9 (36.7)	54.5 (43.0)	45.8 (43.8)
Prior statin prescribing, %^a^	64.4 (10.8)	63.1 (9.6)	63.1 (10.8)

^a^ Percentage of all patients eligible for a statin who were prescribed one prior to the trial.

**Table 2.** Characteristics of the Patient Sample

Characteristic	No. (%)		
	Usual Care (n = 1566)	Active Choice (n = 1743)	Active Choice With Peer Comparison (n = 1465)
Sociodemographics			
Age, mean (SD), y	62.1 (8.5)	62.4 (8.2)	62.5 (8.2)
Male	828 (52.9)	1033 (59.3)	764 (52.2)
Race/Ethnicity			
White non-Hispanic	1004 (64.1)	1110 (63.7)	926 (63.6)
Black non-Hispanic	420 (26.8)	479 (27.5)	419 (28.6)
Other	142 (9.1)	154 (8.8)	120 (8.2)
Insurance			
Private	895 (57.2)	981 (56.3)	803 (54.8)
Medicare	611 (39.0)	684 (39.2)	602 (41.1)
Medicaid	60 (3.8)	78 (4.5)	60 (4.1)
Annual household income, $^a^			
<50 000	435 (27.8)	563 (32.3)	406 (27.7)
50 000-100 000	784 (50.1)	960 (55.1)	733 (50.0)
>100 000	341 (21.8)	206 (11.8)	312 (21.3)
Missing	6 (0.4)	14 (0.8)	14 (1.0)
Baseline measures			
ASCVD risk score, mean (SD)	13.2 (7.9)	14.1 (8.4)	13.3 (8.0)
Body mass index, mean (SD)^b^	30.5 (7.0)	30.6 (7.0)	30.1 (6.5)
Charlson comorbidity index score, median (IQR)	0 (0-1)	0 (0-1)	0 (0-1)
LDL-C level, mean (SD), mg/dL	116.8 (31.5)	117.1 (30.1)	114.3 (28.9)
Clinical ASCVD	223 (14.2)	209 (12.0)	201 (13.7)
Congestive heart failure	51 (3.3)	45 (2.6)	49 (3.3)
Diabetes	417 (26.6)	435 (25.0)	349 (23.8)
Hypertension	860 (54.9)	1052 (60.4)	857 (58.5)
Smoking history	645 (41.2)	764 (43.3)	640 (43.7)

Abbreviations: ASCVD, atherosclerotic cardiovascular disease; IQR, interquartile range; LDL-C, low-density lipoprotein cholesterol.

SI conversion factor: To convert LDL-C to millimoles per liter, multiply by 0.0259.

^a^ Median household income from the US Census Bureau based on patient’s zip code.

^b^ Body mass index calculated as weight in kilograms divided by height in meters squared.

In the active choice arm, 16 of 32 PCPs (50.0%) accessed the patient dashboard, and 4 of 32 (12.5%) submitted statin prescriptions through the dashboard, but only 2 of 32 (6.3%) signed statin prescription orders in the EHR. In the active choice with peer comparison arm, 12 of 32 PCPs (37.5%) accessed the patient dashboard, 8 of 32 (25.0%) submitted statin prescriptions through the dashboard, and 8 of 32 (25.0%) signed statin prescription orders in the EHR.

During the intervention, the percentage of patients prescribed a statin was 2.6% (40 of 1566) in the usual care arm, 6.7% (116 of 1743) in the active choice arm, and 8.0% (117 of 1465) in the active choice with peer comparison arm. In the main adjusted model, compared with usual care, there was a significant difference in statin prescribing in the active choice with peer comparison arm (adjusted difference in percentage points, 5.8; 95% CI, 0.9-13.5; *P* = .008), but not in the active choice arm (adjusted difference in percentage points, 4.1; 95% CI, −0.8 to 13.1; *P* = .11). These findings were similar in sensitivity analyses that also adjusted for patient characteristics and PCP panel size (Table 3).

**Table 3.** Statin Prescription Outcomes

Variable	Usual Care	Active Choice	Active Choice With Peer Comparison
Unadjusted			
Patients prescribed a statin, No./total patients, No. (%)	40/1566 (2.6)	116/1743 (6.7)	117/1465 (8.0)
*P* value	NA	<.001	<.001
Main model^a^			
Percentage point difference relative to usual care (95% CI)	NA	4.1 (−0.8 to 13.1)	5.8 (0.9 to 13.5)
*P* value	NA	.11	.008
Main model also adjusted by patient characteristics and PCP panel size^b^			
Percentage point difference relative to usual care (95% CI)^c^	NA	4.0 (−1.0 to 12.9)	5.8 (0.7 to 13.5)
*P* value	NA	.11	.02

Abbreviations: NA, not applicable; PCP, primary care physician.

^a^ Main model used generalized estimating equations with a logit link and an independence correlation structure using PCP as the clustering unit.

^b^ Patient characteristics in the sensitivity model include age, sex, race/ethnicity, insurance type, median annual household income by zip code, Charlson comorbidity index score, body mass index, most recent low-density lipoprotein cholesterol level, 10-year atherosclerotic cardiovascular disease risk score, clinical atherosclerotic cardiovascular disease, congestive heart failure, diabetes, hypertension, and smoking history.

^c^ Percentage point differences obtained using the bootstrap procedure.

In exploratory subset analyses, black race and Medicaid insurance were associated with greater rates of statin prescription in the intervention arms. Among black patients, the percentage of patients prescribed a statin was 1.2% (5 of 420) in the usual care arm, 16.3% (78 of 479) in the active choice arm, and 16.0% (67 of 419) in the active choice with peer comparison arm. Among patients with Medicaid, the percentage of patients prescribed a statin was 1.7% (1 of 60) in the usual care arm, 15.4% (12 of 78) in the active choice arm, and 15.0% (9 of 60) in the active choice with peer comparison arm. These differences are likely owing to greater use of the active choice dashboard by PCPs whose patient panels included greater proportions of patients who were black and/or had Medicaid insurance (eTable 1, eTable 2, and eTable 3 in Supplement 2). In adjusted models including interaction terms for race/ethnicity and insurance and adjusting for PCP degree, results were similar to the main adjusted model (eTable 4 in Supplement 2). No adverse events were reported during the trial.

## Discussion

Providing physicians with an automated dashboard of patients eligible for statins was most effective at increasing statin prescription rates when using nudges that combined active choice framing in the dashboard with a 1-time email including peer comparison feedback on performance. The same intervention without peer comparison feedback resulted in statin prescription rates that were higher than usual care, but there was not enough power to detect a statistically significant difference.

Our findings reveal several important implications for future intervention design. First, our findings add to the growing literature on using patient dashboards to change physician behavior. A trial by Rat et al^29^ generated a list of patients due for colorectal cancer screening and found that sending the list to physicians led to a significant increase in screening. However, the patient list was sent by mail and may be less scalable than approaches that leverage technology. The list was also not actionable without manual effort from the physician or practice. The intervention in our study was conducted outside of the EHR to test its effect and optimize the design. However, future studies could test ways to implement an active choice patient dashboard within the EHR to better integrate within clinicians’ workflow.

Second, a key element of our intervention design was the use of insights from behavioral economics to design choice architecture, specifically through active choice framing. In prior observational studies,^16,17^ we found that active choice could be used to increase clinician ordering of preventive services, including influenza vaccination, colonoscopy, and mammography. In this trial, physicians could review patients using the automated patient dashboard and make decisions on statin prescriptions, either in bulk or individually. By offering multiple options to prescribe a guideline-indicated statin and requiring a reason to say no, clinicians may be nudged toward prescribing a statin.^30,31^ The pragmatic manner in which these approaches were implemented demonstrates how a learning health care system could take a systematic approach to testing nudges in health care.^32^

Third, peer comparison feedback delivered just once by email was effective at increasing physician engagement with the automated patient dashboard. Compared with active choice alone, the addition of peer comparison feedback increased the number of physicians submitting prescriptions through the dashboard (25% vs 13%) and the number who actually prescribed statins through the EHR (25% vs 6%). A trial by Meeker et al^19^ that tested behavioral interventions to lower unnecessary antibiotic prescribing also found that socially motivated interventions, such as accountable justification and peer comparison, were successful, but suggested alternatives—an approach that was not socially motivated—were not. Despite the differences in statin prescription rates in our study, physician engagement with the dashboard was low. Informal feedback from physicians suggested this may have been because notifications came from the research team rather than clinical leadership or practice managers. In addition, the intervention was low touch in that the physicians received only 1 email with up to 2 reminders. These issues could be addressed in future studies that compare different designs or frequencies of peer comparison feedback and their effect on prescribing behaviors.

Fourth, health care systems are looking for strategies to better manage populations of patients. We enabled physicians to prescribe statins outside of traditional office visits with the patient. This approach is proactive in that it does not require waiting for a patient to come to the clinic to make a decision. It also might optimize future clinic visits by removing the identification of eligible patients and process of prescribing so that the physician and patient can focus more on other aspects of care. It is also relatively low cost and therefore more scalable than other approaches. However, the approach does present some challenges. Physicians with larger patient panels may face difficulties managing these types of decisions outside of their traditional clinic model when they receive a long list of eligible patients at 1 time without additional support. Future interventions could consider a drip model in which physicians are delivered peer comparison feedback monthly over a longer period, thereby providing multiple opportunities to address gaps in care for smaller subsets of patients.

While the effects were statistically significant, they were modest. These interventions would likely fit better within physician workflow if deployed through the EHR rather than alongside it. Indeed, we had wanted to create the dashboard in such a way that making a decision for a patient would automatically generate and sign a prescription order through the EHR. However, by first testing different forms of nudges, we could optimize the design of the intervention before implementing and scaling it within the EHR, which involves the additional time and expense of programming new functionality. Had that connection been seamless, as it would be in a more integrated system, statin prescription rates would likely have been higher.

### Limitations

Our study had limitations. First, physicians and patients were from a single health care system, which may limit generalizability. However, our sample included 32 different practice sites from 2 states. Second, we did not inform physicians in the control arm that they were part of a trial. While in other settings one might want to control for the Hawthorne effect, in this case the potentially greater sense of being observed in the intervention group reflects the reality of these interventions in their practical application outside of research settings and so the result is a more pragmatic comparison. Third, it is possible that some patients were previously prescribed a statin at another health care system that was not captured by our EHR; however, since the randomization was well balanced, it is likely that these types of patients were also balanced across arms. None of the patients in the trial were already actively taking a statin. Fourth, we evaluated statin prescription rates but did not have data on prescription fill rates. Fifth, given the study design, we were unable to isolate the effects of the peer comparison feedback alone. Sixth, our intervention was 2 months in duration, and further study is needed to assess the effects of longer interventions.

## Conclusions

We found that an automated patient dashboard using nudges that combined active choice framing with peer comparison feedback led to a modest but significant increase in statin prescribing rates. Our findings demonstrate the potential of using insights from behavioral economics in highly automated systems to nudge physicians toward guideline-concordant prescribing.

## Notes

Supplement 1. **Trial Protocol**

Supplement 2. **eFigure 1.** Active Choice Intervention Dashboard (example 1) **eFigure 2.** Active Choice Intervention Dashboard (example 2) **eFigure 3.** Email to PCPs in Active Choice Arm **eFigure 4.** Email to PCPs in Active Choice With Peer Comparison Arm **eFigure 5.** Letter to Patients About Statin Prescription **eTable 1.** Proportion of Each PCP’s Eligible Panel and Statin Prescriptions That Were for Black and Medicaid Patients (usual care arm) **eTable 2.** Proportion of Each PCP’s Eligible Panel and Statin Prescriptions That Were for Black and Medicaid Patients (active choice arm) **eTable 3.** Proportion of Each PCP’s Eligible Panel and Statin Prescriptions That Were for Black and Medicaid Patients (active choice with peer comparison arm) **eTable 4.** Statin Prescription Outcomes in Adjusted Models With PCP Degree and Interaction Terms for Race and Insurance
