# Supplementary material for: Effect of an Automated Patient Dashboard Using Active Choice and Peer Comparison Performance Feedback to Physicians on Statin Prescribing: The PRESCRIBE Cluster Randomized Clinical Trial
Source: JAMA Netw Open. 2018 Jul 27;1(3):e180818. doi: 10.1001/jamanetworkopen.2018.0818 (PMC6324300; doi:10.1001/jamanetworkopen.2018.0818)
Supplement: Supplement 1. — Trial Protocol [file jamanetwopen-1-e180818-s001.pdf]

Supplement 1

Study Protocol

Table of Contents

1. Summary of changes to the protocol

2. Study Protocol

Summary of Changes to the Protocol

There were no changes to the study protocol.

Study Protocol

A Randomized, Controlled Trial Using
Active Choice to Promote Statin Prescription Initiation

Study Protocol

January 2017

Outline

1. Abstract

2. Overall objectives

3. Aims

    3.1 Primary outcome

    3.2 Secondary outcomes

4. Background

5. Study design

    5.1 Design

    5.2 Study duration

    5.3 Target population

    5.4 Accrual

    5.5 Key inclusion criteria

    5.6 Key exclusion criteria

6. Subject recruitment

7. Subject compensation

8. Study procedures

    8.1 Consent

    8.2 Procedures

9. Analysis plan

10. Investigators

11. Human research protection

    11.1 Data confidentiality

    11.2 Subject confidentiality

    11.3 Subject privacy

11.4 Data disclosure

11.5 Data safety and monitoring

11.6 Risk/benefit

  11.6.1 Potential study risks

  11.6.2 Potential study benefits

  11.6.3 Risk/benefit assessment

1. Abstract

Cardiovascular (CV) events are the leading cause of mortality in the United States. Statins have been demonstrated to be an effective tool for reducing CV events and mortality, but statins are often not prescribed for patients that meet evidence-based guidelines. In this study, we will evaluate a health system initiative using active choice to identify eligible patients and prompt primary care physicians to initiate statin prescription. In partnership with the health system, this will be conducted as a randomized, controlled trial to evaluate its effect

2. Overall objectives

The objective of the study is to evaluate the effect of a health system initiative using active choice with and without social comparisons feedback to encourage physicians to prescribe statins for patients that meet evidence-based guidelines.

3. Aims

3.1 Primary outcome

The primary outcome measure is the change in percentage of eligible patients prescribed a statin medication within 2 months of intervention completion.

3.2 Secondary outcome

Not applicable

4. Background

Statins, or HMG-CoA reductase inhibitors, are an effective and well tolerated treatment for hyperlipidemia and reduce CV event occurrence and mortality.[1,2] However, despite their demonstrated clinical utility and wide acceptance, statins are underutilized and not prescribed to many patients who would benefit from them under current guidelines.[3] In a recent study looking at the diabetic population, only about 35% of patients who were eligible and lacked contraindications for a statin were prescribed a statin.[4]

In 2013, the American Heart Association and the American College of Cardiology created a new method of estimating 10-year cardiovascular disease (CVD)[5]. They also concurrently released new guidelines for statin prescription that used a combination of age, low-density lipoprotein cholesterol (LDL-C) levels, diabetes diagnosis, and 10-year CVD diagnosis to establish clinical benefit of statins.[6] Although this patient information is readily available to primary care physicians, clinicians are not often presented with the relevant clinical information in a streamlined manner that aids making decisions about a particular guideline.

Active choices, or compelling people to make a decision between alternatives, have been demonstrated to increase compliance to a beneficial intervention and may offer an opportunity to

increase statin prescription intiation.[7,8] Social comparisons feedback has also been demonstrated to change physician behavior in many contexts including antibiotic prescribing.[9]

References

[1] Robinson JG. Accumulating evidence for statins in primary prevention. *JAMA.* 2013;310(22):2405-2406.

[2] Pandya A, Sy S, Cho S, Weinstein MC, Gaziano TA. Cost-effectiveness of 10-Year Risk Thresholds for Initiation of Statin Therapy for Primary Prevention of Cardiovascular Disease. *JAMA.*2015;314(2):142-150.

[3] Hirsh, B. J., Smilowitz, N. R., Rosenson, R. S., Fuster, V., and Sperling, L. S. (2015). Utilization of and adherence to guideline-recommended lipid-lowering therapy after acute coronary syndrome: opportunities for improvement. *J. Am. Coll. Cardiol.* 66, 184–192.

[4] Brandy R. Pauff, Michael R. Jiroutek, Melissa A. Holland, Beth S. Sutton, Statin Prescribing Patterns: An Analysis of Data From Patients With Diabetes in the National Hospital Ambulatory Medical Care Survey Outpatient Department and National Ambulatory Medical Care Survey Databases, 2005–2010, Clinical Therapeutics, Volume 37, Issue 6, 1 June 2015, Pages 1329-1339, http://dx.doi.org/10.1016/j.clinthera.2015.03.020.

[5] 2013 Prevention Guidelines ASCVD Risk Estimator. http://www.acc.org/tools-and-practice-support/mobile-resources/features/2013-prevention-guidelines-ascvd-risk-estimator). Accessed June 30, 2016.

[6] Stone NJ, Robinson JG, Lichtenstein AH, et al; American College of Cardiology/American Heart Association Task Force on Practice Guidelines. 2013 ACC/AHA guideline on the treatment of blood cholesterol to reduce atherosclerotic cardiovascular risk in adults: a report of the American College of Cardiology/American Heart Association Task Force on Practice Guidelines. *Circulation*. 2014;129(25)(suppl 2):S1-S45.

[7] Patel MS, Volpp KG. Leveraging Insights from Behavioral Economics to Increase the Value of Health-Care Service Provision. *Journal of General Internal Medicine*. 2012;27(11):1544-1547. doi:10.1007/s11606-012-2050-4.

[8] Keller AP, Harlam B, Loewenstein G, Volpp KG. Enhanced active choice: A new method to motivate behavior change. J Consum Psychol. 2011;21:376–383.

[9] Meeker D, Linder JA, Fox CR, et al. Effect of Behavioral Interventions on Inappropriate Antibiotic Prescribing Among Primary Care Practices: A Randomized Clinical Trial. JAMA. 2016;315(6):562-70.

5. Study design

5.1 Design

This study will use a randomized, controlled trial to evaluate a health system initiative. The electronic health record will be used to generate lists of patients who meet criteria for statin prescription using the 2013 ACC/AHA (American College of Cardiology / American Heart Association) guidelines. Primary care physicians at the University of Pennsylvania Health System will be randomly assigned to a control group with no intervention or one of two intervention groups as follows:

--Intervention 1: Active choice--

Primary care physicians will be prompted by email to review an online form with a list of their patients who are eligible for statin therapy but have not been prescribed one. Clinical information including the following will be presented for each patient (as available): age, history of atherosclerotic cardiovascular disease (ASCVD) or diabetes, recent test results including lipid profiles and liver function tests. If appropriate information is available, the ASCVD risk score will calculated and displayed.

Using the form, the physician will be able to select atorvastatin 20mg for all of the patients or can individually review information for each patient and select from the following options: 1) prescribe atorvastatin 20mg once daily; 2) prescribe atorvastatin at a different dose (10mg, 40mg or 80mg); 3) prescribe a different statin (choose from simvastatin, pravastatin, or rosuvastatin); 4) do not prescribe at statin and select a reason (patient declined; patient allergy; other).

The online form will be created using a HIPAA compliant website on secure University of Pennsylvania Health System servers. The website will require the physician to sign in using their PennKey and password. Once the form is submitted, a member of the study team will enter the orders in EPIC for the physician to review and sign. The physician will receive notification that these orders have been entered and are ready to review. A letter to the patient will be generated describing the physician's review of their information, prescription of a statin, and the risks and benefits of taking a statin medication.

--Intervention 2: Active choice and social comparisons—

The procedure listed above in intervention 1 will be used. In addition, physicians in this arm will receive social comparisons feedback on their statin prescribing behavior as follows: Those below the median will be told the statin prescribing rate for the average (50th percentile). Those above the median but below the top performers will only be told the prescribing rate of the top performers. Those above the 90th percentile will be told they are a top performer.

5.2 Study duration

The study is expected to begin in February 2017 and take one year to complete.

5.3 Target population

Patients followed by primary care physicians at the University of Pennsylvania Health System who using evidence-based guidelines are eligible to be prescribed a statin.

5.4 Accrual

Primary care physicians at the University of Pennsylvania Health System will be randomly assigned to one of the three arms. Based on an initial data review, we have made the following assumptions: baseline statin initiation rate is about 65%, median number of patients eligible but not on a statin per primary care physician is 38, intracluster correlation for statin prescribing is 0.026. Given these baseline, we estimate that a sample size of 84 primary care physicians (28 per arm) will provide at least 90% power to detect a 10% difference between each intervention arm and the control arm, using a Bonferroni correction to define an alpha of .017 (three comparisons) as our threshold for statistical significance.

5.5 Key inclusion criteria

Patients must meet the following criteria to be eligible for the study:

1) Have a primary care physician at the University of Pennsylvania Health System; 2) Meets American College of Cardiology / American Heart Association (ACC/AHA) guidelines for statin prescription as follows: a) Any form of atherosclerotic cardiovascular disease (ASCVD); b) LDL-C levels of 190 mg/dL or greater on their most recent lipid panel; c) Age 40 to 75 years with diabetes and LDL-C levels of 70-189 mg/dL on their most recent lipid panel; d) Age 40 to 75 years with 10-year ASCVD risk of 7.5% or greater.

5.6 Key exclusion criteria

Patients will be excluded if any of the following criteria is met: 1) Allergy to statins; 2) Severe renal insufficiency defined as glomerular filtration rate (GFR) less than 30 mL/min; 3) Adverse reaction to statins including a) myopathy, ICD-10 G72.0 G72.9, ICD-9 3594 3599; b) Rhabdomyolysis, ICD-10 M62.82, ICD-9 72888; c) hepatitis, ICD-10 K716, ICD-9 5733

Physicians (and their respective patients) will be excluded if they have less than 10 patients among their entire panel that are eligible for a statin medication.

6. Subject recruitment

Information on primary care physicians and their patients at the University of Pennsylvania Health system will be obtained from the electronic health record using Penn Data Store and Clarity, an EPIC reporting database.

7. Subject compensation

No compensation will be offered in this study.

8. Study procedures

8.1 Consent

A waiver of informed consent is requested. This is a health system initiative that will be implemented. The study is to evaluate that initiative. Therefore, physicians and their patients will not be consented as this is the standard of practice per the health system initiative. Without a waiver of the consent, the initiative would still be implemented by the health system, but the study would be infeasible. There are several additional reasons why we feel a waiver of consent should be granted. First, it is not feasible to consent every patient and physician and as mentioned this initiative would occur with or without the study of it. Second, if members of the control group were consented, they would know they were being studied and this could change their behavior. This could potential disrupt the design of the study and making interpretation of the findings challenging. Third, physicians are not being forced to prescribe statins for their patients. Instead, they are being reminded of evidence-based guidelines and offered an opportunity to review pertinent information and prescribe a statin. This is no different than standard of care in which a physician would review the same information and decide to prescribe a statin or not. The initiative is simply a reminder for the physician and makes their standard of care process easier to conduct.

8.2 Procedures

Data on primary care physicians and their patients at the University of Pennsylvania Health System will be obtained from Penn Data Store and Clarity (Epic's data reporting database). Physician data includes demographic information (age, race, gender, type of medical degree, etc.) and may be also obtained from publicly available databases or websites online. Patient information includes demographic information, information about comorbid conditions (including diabetes, hypertension, and chronic kidney disease, and comorbid conditions needed to calculate the Charleston Comorbidity Index, laboratory test results (total cholesterol, triglycerides, LDL-C, HDL-C, liver function tests, creatinine, and glomerular filtration rate), and any contraindications to statin prescription (allergies or history of adverse reactions).

After identifying eligible physicians and patients, block randomization will occur at the physician level using block sizes of three and stratifying by quartile of baseline statin prescribing rate.

9. Analysis plan

In the main analysis, we will perform unadjusted comparisons of the change in the percent of eligible patients that are prescribed a statin before and after the intervention by study group using chi-square tests. To evaluate the robustness of the findings, a multivariate logistic regression

model will be fit adjusting for baseline statin prescription status as well as patient and physician factors. Binary variables will be created for time (pre- and post-intervention), study arm (control or intervention), and statin prescription (no or yes). The outcome of interest will be the interaction between the binary variables time, study arm, and statin prescription. All hypothesis tests will be two-sided using an alpha of 0.017 as our threshold for statistical significance. Clustering will be conducted at the level of the physician.

10. Investigators

Mitesh Patel, MD, MBA, MS is the Principal Investigator (PI) and is an Assistant Professor of Medicine and Health Care Management at the Perelman School of Medicine and The Wharton School at the University of Pennsylvania. He has past experience leading clinical trials to deploy interventions impacting patient care and outcomes. He currently spends 80% of his effort on research and 20% on clinical and teaching activities.

David Asch, MD, MBA is a co-investigator and is a Professor of Medicine and a Professor of Health Care Management at the Perelman School of Medicine and The Wharton School at the University of Pennsylvania and the Executive Director at the Center for Health Care Innovation at the University of Pennsylvania. He has extensive past experience conducting research, designing and leading clinical trials, and implementing new policies to improve patient care.

11. Human research protection

11.1 Data confidentiality

Computer-based files will only be made available to personnel involved in the study through the use of access privileges and passwords. Wherever feasible, identifiers will be removed from study-related information. Precautions are already in place to ensure the data are secure by using passwords and HIPAA-compliant encryption.

11.2 Subject confidentiality

Data on physicians and patients will be obtained from Epic and Penn Data Store. Any information that is obtained will be used for research purposes only. Information on patients will only be disclosed within the study team and to the patient’s primary care physician. All study staff will be reminded of the confidential nature of the data collected and contained in these databases.

The Leonard Davis Insitute’s Health Services Research Data Center (LDI HSRDC) will be the hub for the hardware and database infrastructure that will support the statistical analysis. The LDI HSRDC is a secure computing environment for a large volume of highly sensitive data. All members that access data must complete HIPAA training including secure data transfer,

passwords, computer security habits and knowledge of what constitutes misuse or inappropriate use of the server. Only trained study staff will have access to the code that links the unique identifier to the subject's identity. Electronic data will be stored on secure, password-protected firewalled servers at the University of Pennsylvania.

11.3 Subject privacy

All efforts will be made by study staff to ensure subject privacy. Data will be evaluated in a de-identified manner whenever possible.

11.4 Data disclosure

Information on patients will only be disclosed within the study team and to the patient's primary care physician (to whom this information is already available).

11.5 Data safety and monitoring

The investigator will provide oversight for the study evaluation of this health system initiative. Physician practices will follow their standards of care to manage patients initiated on statins.

11.6 Risk/benefit

11.6.1 Potential study risks

The potential risks associated with this study are minimal. Breach of data is a potential risk that will be mitigated by using HIPAA compliant and secure data platforms for the intervention (Penn Medicine servers) and evaluation (LDI HSRDC server). Statins do have known potential side effects, mostly commonly myopathy. To reduce this risk, patients with document allergies or adverse events will be excluded from the initiative and study. Patients with renal failure will also be excluded as they may require lower doses of statins than patients without renal failure. Prescribing statins and monitoring for known side effects are otherwise standard of care within primary care practices.

11.6.2 Potential study benefits

Statins have been demonstrated as an effective tool for reducing cardiovascular events. Early intervention with statins can potentially be life saving for patients who are at risk or already have a diagnosis of cardiovascular disease. An intervention that prompts physicians to actively think about evidence-based guidelines and high-value treatment could contribute positively to patient care and outcomes. However, it is possible that patients will receive no benefit from this study.

11.6.3 Risk/benefit assessment

The risk/benefit ratio is highly favorable given the potential benefit from eligible patients being prescribed a statin, that prescription and monitoring of a statin is within the standards of care in a

primary care practice, and that efforts have been put into place to minimize the risk of breach of data.
