# Supplementary material for: Effect of an Automated Patient Dashboard Using Active Choice and Peer Comparison Performance Feedback to Physicians on Statin Prescribing: The PRESCRIBE Cluster Randomized Clinical Trial
Source: JAMA Netw Open. 2018 Jul 27;1(3):e180818. doi: 10.1001/jamanetworkopen.2018.0818 (PMC6324300; doi:10.1001/jamanetworkopen.2018.0818)
Supplement: Supplement 2. — eFigure 1. Active Choice Intervention Dashboard (example 1) eFigure 2. Active Choice Intervention Dashboard (example 2) eFigure 3. Email to PCPs in Active Choice Arm eFigure 4. Email to PCPs in Active Choice With Peer Comparison Arm eFigure 5. Letter to Patients About Statin Prescription eTable 1. Proportion of Each PCP’s Eligible Panel and Statin Prescriptions That Were for Black and Medicaid Patients (usual care arm) eTable 2. Proportion of Each PCP’s Eligible Panel and Statin Prescriptions That Were for Black and Medicaid Patients (active choice arm) eTable 3. Proportion of Each PCP’s Eligible Panel and Statin Prescriptions That Were for Black and Medicaid Patients (active choice with peer comparison arm) eTable 4. Statin Prescription Outcomes in Adjusted Models With PCP Degree and Interaction Terms for Race and Insurance [file jamanetwopen-1-e180818-s002.pdf]

Supplementary Online Content

Patel MS, Kurtzman GW, Kannan S, et al. Effect of an automated patient dashboard using active choice and peer comparison performance feedback to physicians on statin prescribing: the PRESCRIBE cluster randomized clinical trial. *JAMA Netw Open*. 2018;1(3):e180818. doi:10.1001/jamanetworkopen.2018.0818

eFigure 1. Active Choice Intervention Dashboard (example 1)

eFigure 2. Active Choice Intervention Dashboard (example 2)

eFigure 3. Email to PCPs in Active Choice Arm

eFigure 4. Email to PCPs in Active Choice With Peer Comparison Arm

eFigure 5. Letter to Patients About Statin Prescription

eTable 1. Proportion of Each PCP's Eligible Panel and Statin Prescriptions That Were for Black and Medicaid Patients (usual care arm)

eTable 2. Proportion of Each PCP's Eligible Panel and Statin Prescriptions That Were for Black and Medicaid Patients (active choice arm)

eTable 3. Proportion of Each PCP's Eligible Panel and Statin Prescriptions That Were for Black and Medicaid Patients (active choice with peer comparison arm)

eTable 4. Statin Prescription Outcomes in Adjusted Models With PCP Degree and Interaction Terms for Race and Insurance

This supplementary material has been provided by the authors to give readers additional information about their work.

© 2018 Patel MS et al. *JAMA Network Open*.

eFigure 1. Active Choice Intervention Dashboard (example 1)

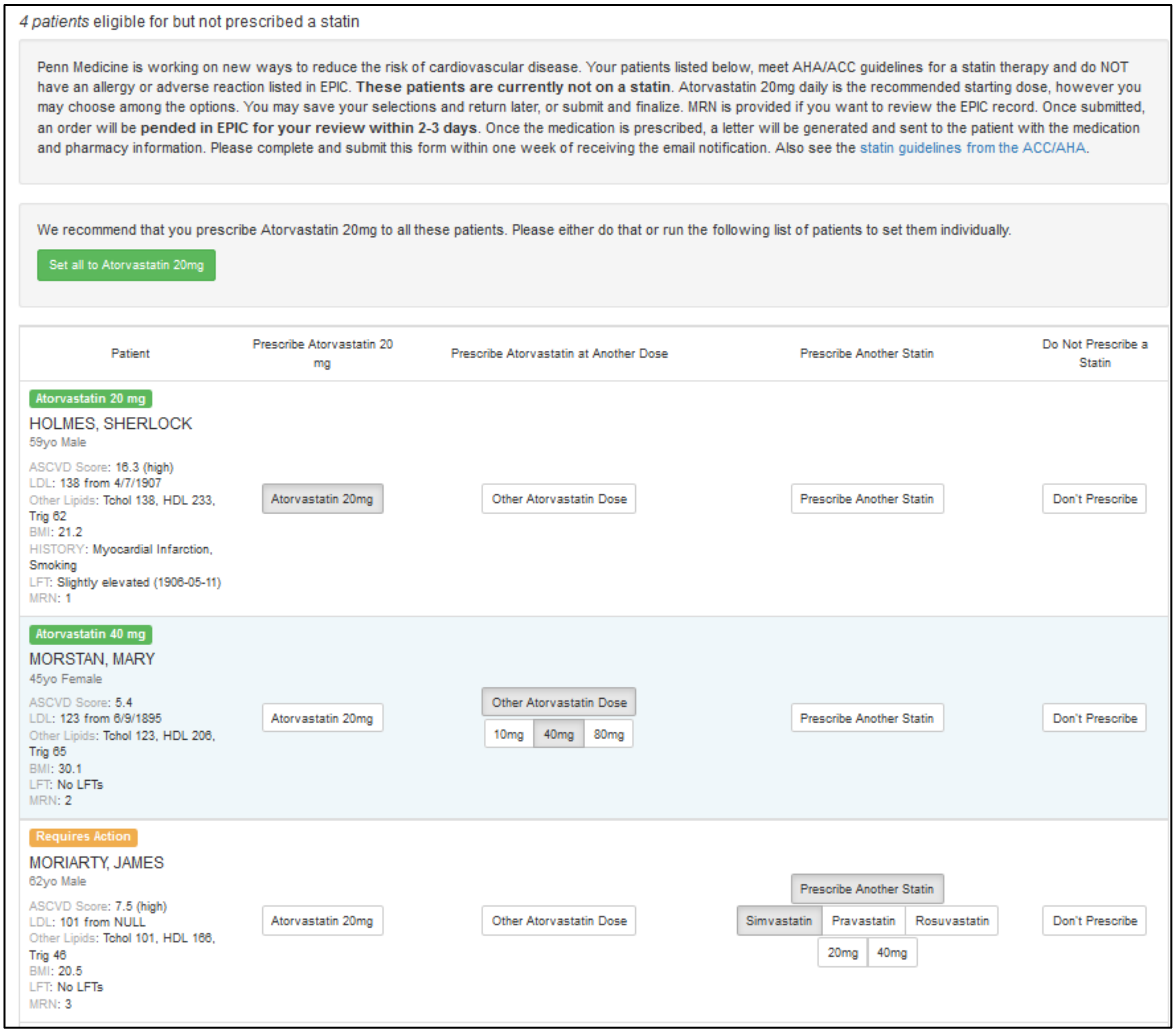

*No real patient information is displayed. This figure is for illustrative purposes only

© 2018 Patel MS et al. *JAMA Network Open*.

eFigure 2. Active Choice Intervention Dashboard (example 2)

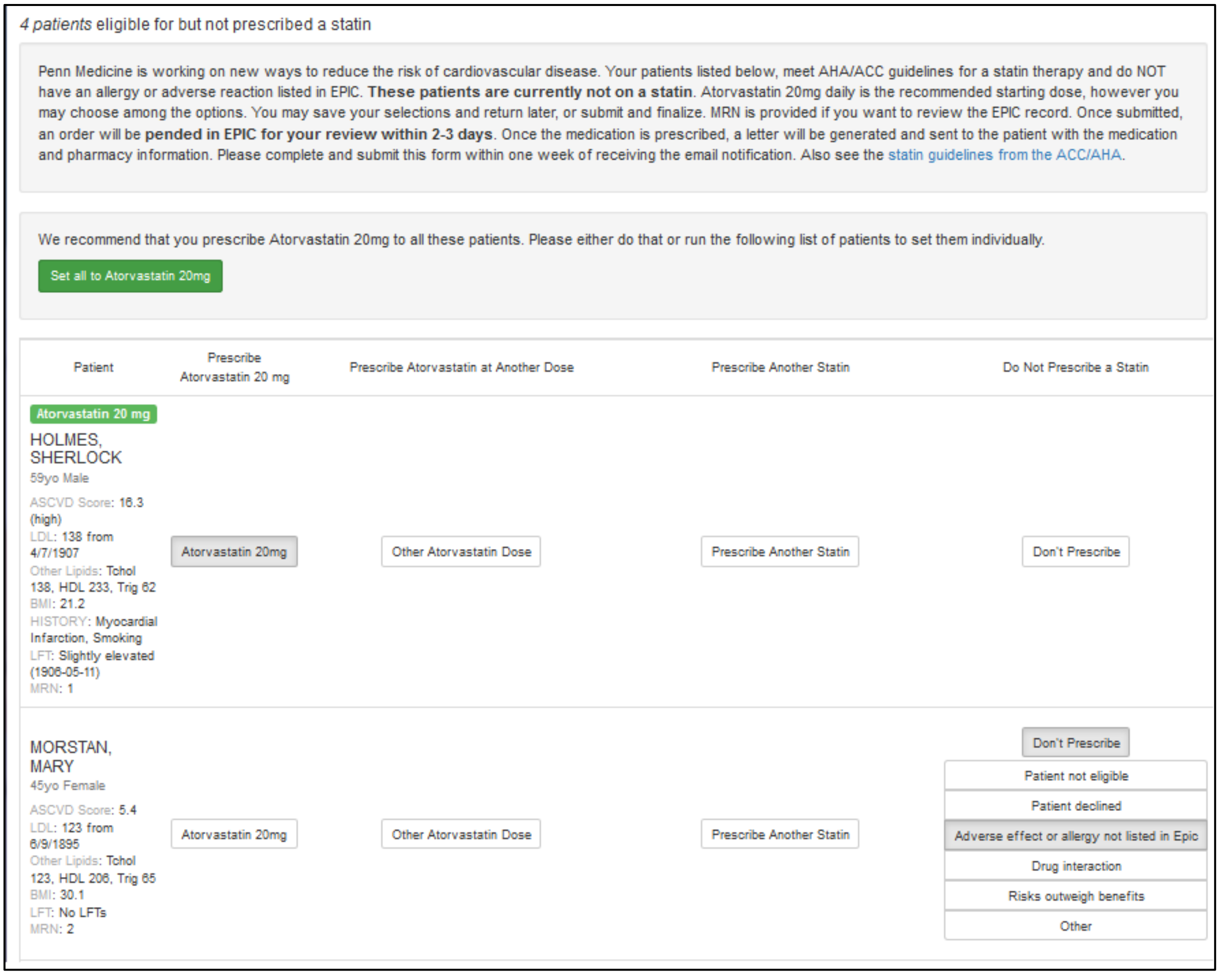

*No real patient information is displayed. This figure is for illustrative purposes only

© 2018 Patel MS et al. *JAMA Network Open*.

eFigure 3. Email to PCPs in Active Choice Arm

Dear Dr.______________,

Penn Medicine is working on new ways to help physicians reduce their patients' risk of cardiovascular disease. Based on AHA/ACC guidelines you have ____ patients who should be on a statin but have not been prescribed one.

You can review a list of your patients and select a statin to start them on here: [insert URL].
You will need UPHS credentials and to be connected to the UPHS Network to access the site.

Please complete the online submission form within one week of this email. If you select the first option to start all of your patients on Atorvastatin 20mg daily, it will take less than one minute to complete. Or, you may review each patient individually and make your selection. Once submitted, a research coordinator will pend an order in Epic for you to review. Once a statin prescription is signed, the patient will be sent a letter notifying them of the prescription, its clinical rationale, potential side effects, and pharmacy location.

If you have any questions, please email us at nudges@upenn.edu.

Sincerely,

Greg Kurtzman
Clinical Research Coordinator, Penn Medicine Nudge Unit

Mitesh Patel, MD
Director, Penn Medicine Nudge Unit

© 2018 Patel MS et al. *JAMA Network Open*.

eFigure 4. Email to PCPs in Active Choice With PCP Degree and Peer Comparison Arm

Dear Dr.______________,

Penn Medicine is working on new ways to help physicians reduce their patients' risk of cardiovascular disease. Based on AHA/ACC guidelines you have ____ patients who should be on a statin but have not been prescribed one.

Among eligible patients, your statin prescribing rate: XX%
[One of the following]
Average of your peers at Penn: XX%
Your top performing peers at Penn: XX%
Among your peer physicians at Penn, you are a top performer. Great job!

You can review a list of your patients and select a statin to start them on here: [insert URL].
You will need UPHS credentials and to be connected to the UPHS Network to access the site.

Please complete the online submission form within one week of this email. If you select the first option to start all of your patients on Atorvastatin 20mg daily, it will take less than one minute to complete. Or, you may review each patient individually and make your selection. Once submitted, a research coordinator will pend an order in Epic for you to review. Once a statin prescription is signed, the patient will be sent a letter notifying them of the prescription, its clinical rationale, potential side effects, and pharmacy location.

If you have any questions, please email us at nudges@upenn.edu.

Sincerely,

Greg Kurtzman
Clinical Research Coordinator, Penn Medicine Nudge Unit

Mitesh Patel, MD
Director, Penn Medicine Nudge Unit

If prescribing rate below the median, show: Average of your peers at Penn: XX%

If prescribing rate between median and 90th percentile, show: Your top performing peers at Penn: XX%

If prescribing rate is 90th percentile or higher, show: Among your peer physicians at Penn, you are a top performer. Great job!

© 2018 Patel MS et al. *JAMA Network Open*.

eFigure 5. Letter to Patients About Statin Prescription

Dear _________________,

This letter is to inform you that Dr.____________ has reviewed your medical record and determined that you would benefit from taking a statin medication to reduce your risk of heart disease.

A 90-day prescription with one refill for ______________________ once daily has been sent to: _________________________________

Statin medications can improve your cholesterol levels and reduce the risk of future heart attack or stroke. Statin medications are generally well-tolerated. While side effects are rare, the most common is muscle aches or soreness. If you experience this or any other changes, please stop taking the medication and call your doctor.

Please pick up this prescription within the next 1-2 weeks and begin taking the medication. If you do not already have an appointment with your primary care physician, please schedule one for within the next 6 months.

Penn Medicine

© 2018 Patel MS et al. *JAMA Network Open*.

eTable 1. Proportion of Each PCP's Eligible Panel and Statin Prescriptions That Were for Black and Medicaid Patients (usual care arm)

Usual Care						
PCP	Enrolled Panel Size			Presribed a Statin		
	N	% Black	% Medicaid	N	% Black	% Medicaid
1	148	2.7	.	10	.	.
2	106	39.6	.	4	.	.
3	67	7.5	1.5	4	.	.
4	68	47.1	2.9	3	66.7	.
5	91	4.4	.	2	.	.
6	100	18.0	.	2	.	.
7	28	.	3.6	2	.	50.0
8	116	5.2	.	2	.	.
9	29	69.0	17.2	2	.	.
10	19	21.1	.	1	100.0	.
11	41	2.4	.	1	.	.
12	38	15.8	5.3	1	.	.
13	33	18.2	6.1	1	.	.
14	52	46.2	9.6	1	100.0	.
15	87	62.1	2.3	1	100.0	.
16	44	.	.	1	.	.
17	22	4.6	.	1	.	.
18	107	12.2	3.7	1	.	.
19	34	41.2	8.8	.	.	.
20	13	46.2	.	.	.	.
21	8	62.5	.	.	.	.
22	28	64.3	.	.	.	.
23	11	27.3	.	.	.	.
24	38	.	2.6	.	.	.
25	74	91.9	23.0	.	.	.
26	37	91.9	18.9	.	.	.
27	13	76.9	7.7	.	.	.
28	33	21.2	6.1	.	.	.
29	7	42.9	28.6	.	.	.
30	20	.	10.0	.	.	.
31	13	76.9	7.7	.	.	.
32	41	4.9	.	.	.	.

*N refers to the patient sample size

© 2018 Patel MS et al. *JAMA Network Open*.

eTable 2. Proportion of Each PCP's Eligible Panel and Statin Prescriptions That Were for Black and Medicaid Patients (active choice arm)

Active Choice						
PCP	Enrolled Panel Size			Presribed a Statin		
	N	% Black	% Medicaid	N	% Black	% Medicaid
1	79	89.9	8.9	66	90.9	9.1
2	49	57.1	14.3	13	69.2	30.8
3	40	62.5	15.0	4	75.0	50.0
4	166	27.1	.	4	25.0	.
5	73	5.5	1.4	4	25.0	.
6	46	.	2.2	3	.	.
7	29	.	.	3	.	.
8	120	20.0	15.0	2	.	.
9	49	12.2	.	2	.	.
10	81	9.9	4.9	2	.	.
11	19	10.5	.	2	50.0	.
12	118	2.5	1.7	2	.	.
13	125	2.4	0.8	2	.	.
14	78	73.1	9.0	2	100.0	.
15	56	87.5	7.1	1	.	.
16	59	6.8	1.7	1	.	.
17	21	61.9	14.3	1	.	.
18	35	48.6	5.7	1	100.0	.
19	9	44.4	.	1	.	.
20	14	.	.	.	.	.
21	143	1.4	1.4	.	.	.
22	19	10.5	.	.	.	.
23	9	88.9	.	.	.	.
24	4	75.0	25.0	.	.	.
25	96	46.9	.	.	.	.
26	14	35.7	.	.	.	.
27	35	11.4	8.6	.	.	.
28	9	77.8	.	.	.	.
29	22	72.7	.	.	.	.
30	58	5.2	5.2	.	.	.
31	20	10.0	10.0	.	.	.
32	48	39.6	6.3	.	.	.

* N refers to the patient sample size

© 2018 Patel MS et al. *JAMA Network Open*.

eTable 3. Proportion of Each PCP's Eligible Panel and Statin Prescriptions That Were for Black and Medicaid Patients (active choice with peer comparison arm)

Active Choice with Peer Comparison						
PCP	Enrolled Panel Size			Presribed a Statin		
	N	% Black	% Medicaid	N	% Black	% Medicaid
1	48	75.0	2.1	33	72.7	3.0
2	46	95.7	10.9	25	96.0	12.0
3	11	90.9	27.3	9	100.0	22.2
4	15	.	.	6	.	.
5	218	8.7	2.3	5	.	.
6	11	9.1	.	4	.	.
7	6	83.3	16.7	4	100.0	25.0
8	4	75.0	.	4	75.0	.
9	89	6.7	1.1	4	.	.
10	80	3.8	1.3	3	.	.
11	15	73.3	20.0	3	66.7	33.3
12	48	43.8	4.2	3	.	.
13	80	2.5	1.3	2	.	.
14	39	87.2	2.6	2	.	.
15	104	2.9	1.9	2	.	.
16	50	16.0	14.0	2	.	.
17	1	100.0	100.0	1	100.0	100.0
18	48	8.3	.	1	.	.
19	41	12.2	.	1	.	.
20	82	56.1	2.4	1	.	.
21	83	7.2	.	1	.	.
22	22	4.5	9.1	1	.	.
23	2	100.0	.	.	.	.
24	6	.	.	.	.	.
25	93	86.0	9.7	.	.	.
26	35	48.6	14.3	.	.	.
27	29	6.9	.	.	.	.
28	36	47.2	.	.	.	.
29	69	29.0	7.2	.	.	.
30	18	22.2	.	.	.	.
31	9	77.8	11.1	.	.	.
32	27	3.7	7.4	.	.	.

* N refers to the patient sample size

© 2018 Patel MS et al. *JAMA Network Open*.

eTable 4. Statin Prescription Outcomes in Adjusted Models With PCP Degree and Interaction Terms for Race and Insurance

	Adjusted Odds Relative to Control (95% CI)	Adjusted Percentage Point Difference Relative to Control (95% CI)	P Value
**Main Model**			
Active Choice	2.7 (0.8, 9.4)	4.1 (-0.8, 13.1)	0.11
Active Choice with Peer Comparisons	3.3 (1.4, 8.0)	5.8 (0.9, 13.5)	<.01
**Main Model Adjusted for Patient Characteristics and PCP Panel Size**			
Active Choice	2.8 (0.8, 10.3)	4.0 (-1.0, 12.9)	0.11
Active Choice with Peer Comparisons	3.5 (1.3, 9.2)	5.8 (0.7, 13.5)	0.01
**Main Model Adjusted for Patient Characteristics, PCP Panel Size, PCP Degree and Interactions for Race and Insurance**			
Active Choice	4.1 (1.3, 12.9)	4.0 (-1.0, 12.9)	0.02
Active Choice with Peer Comparisons	6.0 (2.2, 16.7)	5.8 (0.7, 13.5)	<.001

© 2018 Patel MS et al. *JAMA Network Open*.
